# Engineered Mott ground state in a LaTiO_3+*δ*_/LaNiO_3_ heterostructure

**DOI:** 10.1038/ncomms10418

**Published:** 2016-01-21

**Authors:** Yanwei Cao, Xiaoran Liu, M. Kareev, D. Choudhury, S. Middey, D. Meyers, J.-W. Kim, P. J. Ryan, J.W. Freeland, J. Chakhalian

**Affiliations:** 1 Department of Physics, University of Arkansas, Fayetteville, Arkansas 72701, USA; 2 Department of Physics, Indian Institute of Technology Kharagpur, Kharagpur 721302, India; 3 Advanced Photon Source, Argonne National Laboratory, Argonne, Illinois 60439, USA

## Abstract

In pursuit of creating cuprate-like electronic and orbital structures, artificial heterostructures based on LaNiO_3_ have inspired a wealth of exciting experimental and theoretical results. However, to date there is a very limited experimental understanding of the electronic and orbital states emerging from interfacial charge transfer and their connections to the modified band structure at the interface. Towards this goal, we have synthesized a prototypical superlattice composed of a correlated metal LaNiO_3_ and a doped Mott insulator LaTiO_3+*δ*_, and investigated its electronic structure by resonant X-ray absorption spectroscopy combined with X-ray photoemission spectroscopy, electrical transport and theory calculations. The heterostructure exhibits interfacial charge transfer from Ti to Ni sites, giving rise to an insulating ground state with orbital polarization and *e*_*g*_ orbital band splitting. Our findings demonstrate how the control over charge at the interface can be effectively used to create exotic electronic, orbital and spin states.

## Abstract (web summary)

Interfaces between two dissimilar transition metal oxides can exhibit emergent strongly correlated electronic and magnetic states due to charge transfer and electronic reconfiguration. Here, the authors synthesize and investigate an exotic Mott ground state in LaTiO_3+*δ*_/LaNiO_3_ heterostructures.

## Graphical abstract

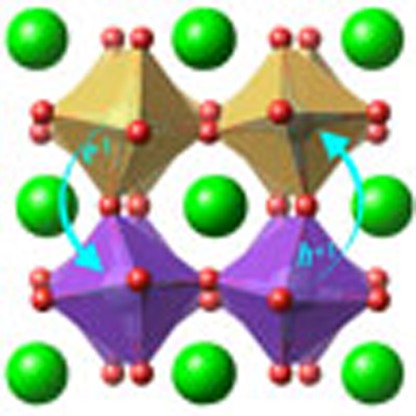

Understanding and controlling the interactions between charge, spin, orbital and structural degrees of freedom in transition-metal oxides is at the centre of modern condensed matter physics^1^,^2^,^3^. In recent years, inspired by the tremendously successful research on physics and applications of ultra-thin semiconductor heterostructures, multilayers of correlated complex oxides have become a platform of choice to generate the emergent electronic and magnetic states, unattainable in the bulk compounds^4^,^5^,^6^,^7^,^8^,^9^,^10^,^11^,^12^,^13^,^14^. The inherent many-body nature of the correlated states, however, raises many fundamental questions that demand experimental validation and expansibility about the applicability of the concepts and formulas developed for semiconductor heterointerfaces^6^. Recent experimental work on complex oxide interfaces^5^,^6^,^7^,^8^,^9^,^10^,^11^,^12^,^13^,^14^ revealed a remarkable importance of the electronic configurations of partially filled *d*-shell transition-metal ions for understanding the emerging many-body phenomena^1^,^2^,^3^, including the Ti 3*d*^1^ configuration in the SrTiO_3_-based two-dimensional electron gases^5^, Cu 3*d*^9^L configuration in the orbitally and magnetically reconstructed states at the manganate-cuprates interfaces (here L denotes a ligand hole on the oxygen ion)^6^,^15^ and Ni 3*d*^8^L configuration in self-doped and orbitally polarized nickelate heterojunctions^2^,^6^,^16^,^17^,^18^,^19^. To induce a specific electronic configuration at the interface, charge transfer (or electron doping) has been proven to be a particularly powerful tool to achieve this goal^20^,^21^,^22^. Even more so than in the doped semiconductors^23^,^24^,^25^,^26^, charge transfer with strong electron–electron correlations and frustrated spin and orbital interactions at the interface may give rise to unexpected collective quantum states not attainable with semiconductor heterojunctions^4^,^5^,^6^. Understanding the mechanism of charge redistribution between layers of Mott materials and the implications of a specific electron reconfiguration arising from the charge transfer is therefore of a great necessity towards the rational design of applications based on strongly correlated electrons^6^,^20^,^21^,^22^.

In semiconductor heterostrutures, the charge transfer can be successfully rationalized in terms of single-electron energy states to profile the energy band bending and the band alignments across the interface^26^,^27^. Following this notion, we recap that within the class of complex oxides with 3*d* electrons there are two types of Mott insulating behaviour parameterized in the Zaanen–Sawatzky–Allen scheme by the relative magnitude of on-site Coulomb repulsion energy *U*_dd_ between *d*-shell electrons versus charge-transfer energy Δ_CT_ between oxygen *p*-shell and the TM *d*-state^28^; based on this, one can distinguish between Mott–Hubbard insulators (MHI, *U*_dd_<Δ_CT_) and charge-transfer insulators or charge-transfer metals (CTM, *U*_dd_>Δ_CT_)^1^,^2^. To date, the vast majority of experimental and theoretical work has been focused on charge doping at the interfaces between MHI and MHI^29^,^30^, and MHI and normal metal^31^,^32^,^33^,^34^. Some of the most remarkable physical phenomena such as high-*T*_C_ superconductivity and colossal magnetoresistance, however, are observed in charge-transfer compounds characterized by the strong hybridization between oxygen 2*p* and transition metal 3*d* states, complex electronic configurations (for example, mixing between *d*^*n*^ and *d*^*n*^L states) and small or even negative charge excitation gap Δ_CT_ (refs 1, 2, 28, 35). In these materials, the role of the lower Hubbard band is replaced by the oxygen states, which in turn implies a very asymmetric physical character for the doped holes (mainly in oxygen levels) and doped electrons (mainly in transition metal *d* levels), for example, correlated metal LaNiO_3_ (LNO). With the original motivation to create a cuprate-like electronic and orbital structures^6^,^36^,^37^, LNO-based perovskite heterostructures have attracted continuous interest^6^,^16^,^17^,^18^,^19^,^20^,^21^,^22^,^38^,^39^,^40^,^41^,^42^,^43^ in spite of the intriguing bulk properties of charge-transfer materials. However, experimentally very little is known about the Mott carrier redistribution and their electronic reconfigurations at the heterointerface between MHI and CTM^20^,^21^,^22^.

Towards this goal, we have synthesized and investigated a prototypical MHI/CTM heterostructure (2 u.c. LaTiO_3+*δ*_/2 u.c. LaNiO_3_) × 10 (2LTO/2LNO thereafter, u.c.=unit cells, *δ*∼0.34 is the concentration of oxygen excess from the ideal Ti^3+^ state). The resulting 2LTO/2LNO heterostructure exhibits an exotic Mott ground state. To quantify this phenomenon, we investigated the interfacial charge transfer from Ti to Ni sites and the reconstruction of the electronic structure by resonant soft X-ray absorption spectroscopy (XAS) at Ti, Ni L_2,3_− and O K-edges combined with X-ray photoemission spectroscopy (XPS), electrical transport and first-principles calculations. X-ray linear dichroism (XLD) spectroscopy was carried out to reveal the orbital polarization and unexpected Ni *e*_*g*_ band splitting. Our findings highlight how the control over charge at the interface can be effectively used to create exotic electronic, orbital and spin states.

## Results

### Interfacial charge transfer

**Figure 1:**
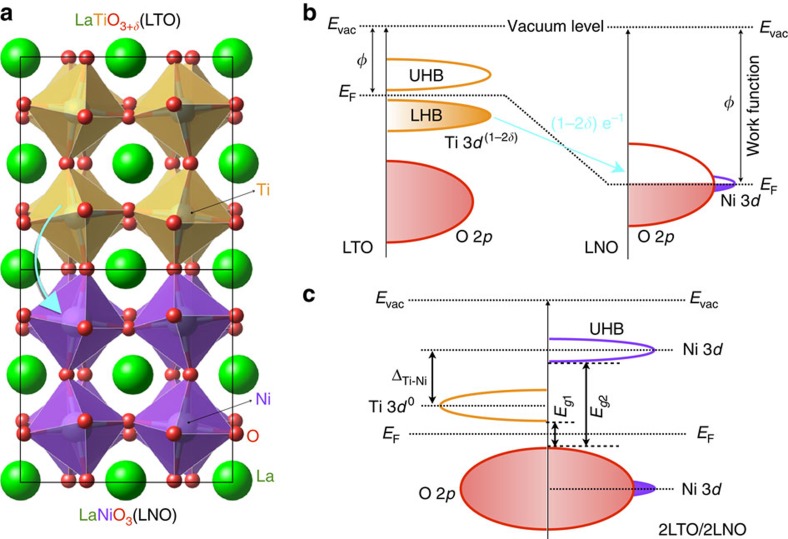
Crystal and band structures of 2LTO/2LNO. (**a**) Schematic view of the atomic arrangement. Sketch of the energy bands before (**b**) and after (**c**) the formation of 2LTO/2LNO interface. The cyan solid arrows in **a**,**b** indicate the direction of interfacial charge transfer (electron, *e*^−1^) from Ti to Ni sites. Here, *E*_*g*1_ (or *E*_*g*2_), charge gap between the highest occupied state (a mixture of oxygen 2*p* and Ni 3*d* states^20^) and the bottom of empty Ti 3*d* (or Ni 3*d*) state; Δ_Ti−Ni_, relative energy difference between empty Ti and Ni 3*d* states; LHB, lower Hubbard band; UHB, upper Hubbard band.

As shown in Fig. 1, driven by the difference between Fermi levels *E*_F_ or chemical potentials in constituent layers across the junction 2LTO/2LNO (see Fig. 1a and Supplementary Fig. 1), in a conventional view the charge redistributes near the interface. As the components of 2LTO/2LNO superlattice (SL), the electronic configuration of CTM LaNi^3+^O_3_ is a mixture of low-spin 3*d*^7^ and high-spin 3*d*^8^L states with the Fermi energy passing though the strongly mixed Ni–O valence states^44^,^45^, whereas as an archetypal MHI (∼0.2 eV gap) bulk LaTi^3+^O_3_ has only one electron (3*d*^1^) occupying the lower Hubbard band^46^,^47^ and its Fermi energy level *E*_F_ is much higher than that of LNO (∼2 eV difference)^48^, as schematically shown in Fig. 1b. By aligning the interfacial bands with respect to the continuing oxygen *p* states on either side of the interface^20^,^49^, the resulting Ti 3*d* band energy position becomes significantly higher than the Fermi energy of the LNO; this in turn implies a one-way charge redistribution from the Ti 3*d* band of LTO into the partially filled Ni *d* and O *p* states of LNO. Recent density functional theory (DFT+*U*) calculations^20^ further tested this naive picture and suggested a full electron charge transfer, that is, Ti *d*^1^+Ni *d*^7^→Ti *d*^0^+Ni *d*^8^ (Fig. 1c). On the other hand, as the experimental electronic configuration of LNO is a mixture of Ni *d*^7^ and *d*^8^L states, the charge transfer may also result in the appearance of additional interfacial electronic states, that is, Ti *d*^1^+Ni *d*^8^L→Ti *d*^0^+Ni *d*^8^ and Ti *d*^1^+Ni *d*^8^L→Ti *d*^0^+Ni *d*^9^L.

**Figure 2:**
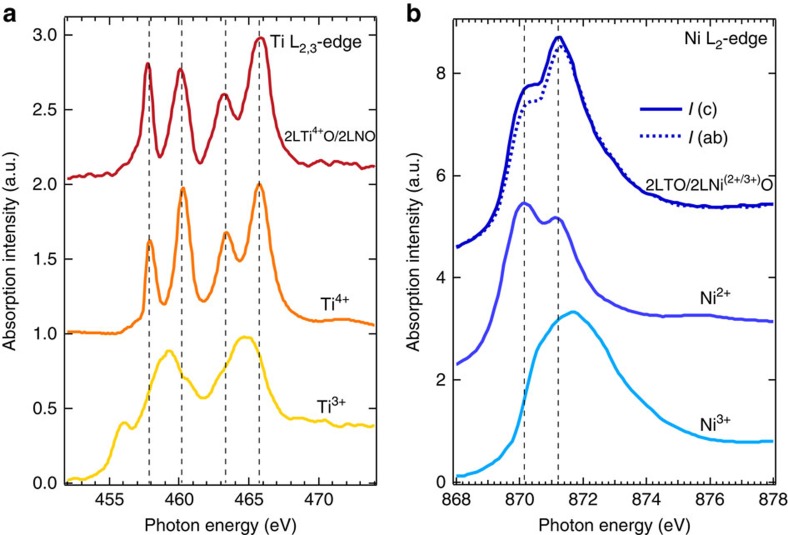
XAS of 2LTO/2LNO. (**a**) Ti L_2,3_-edge. The reference spectra for Ti^4+^ and Ti^3+^ were measured on a SrTi^4+^O_3_ single crystal and YTi^3+^O_3_ film (∼100 nm on TbScO_3_ substrate^59^), respectively. (**b**) Ni L_2,3_-edge. The reference samples are bulk Ni^2+^O and LaNi^3+^O_3_. Out-of-plane (*I*(c), dark blue solid line, *E* || c and *E* is the linear polarization vector of the photon) and in-plane (*I*(ab), dark blue dashed line, *E* || ab) linearly polarized X-ray were used to measure XAS of 2LTO/2LNO at Ni L_2,3_-edge. Black dashed lines are guidelines for peak positions. All spectra were collected and repeated more than two times with bulk-sensitive total fluorescence yield (TFY) detection mode at room temperature.

To investigate the experimental veracity of the theory, we measured the electronic structures of Ti and Ni, to track the charge transfer by element-specific XAS in total fluorescence yield mode (with the bulk probing depth) and by *in-situ* XPS. As seen in Fig. 2a, the features of the Ti L_2,3_-edge in the 2LTO/2LNO sample show excellent agreement with the Ti^4+^ charge and are remarkably different from the spectra of Ti^3+^. This result provides a strong evidence for the occurrence of the charge-transfer Ti *d*^(1−2*δ*)^→Ti *d*^0^ and implies that almost all of the *t*_2*g*_ electrons from Ti sites are transferred elsewhere. The flow of the charge is further verified by the complimentary XAS measurements at the Ni L_2,3_-edge, which clearly shows a strong increase of the Ni charge state, that is, Ni *d*^7^→Ni *d*^(8−2*δ*)^ (see Fig. 2b). A comparison with the bulk reference spectra of Ni^2+^ (double peaks at ∼870.2 and 871.2 eV) and Ni^3+^ (single main peak at ∼871.6 eV) attests that in the 2LTO/2LNO SL the Ni final state is indeed a mixture of Ni^2+^/Ni^3+^ (double peaks), which is also affirmed by the calculated XAS lineshape dependence on the Ni electronic configuration (see Supplementary Fig. 2). To further corroborate these findings, the interfacial charge-transfer phenomenon was studied by measuring the core-level electronic structures of Ti and Ni with *in-situ* XPS (see Supplementary Fig. 3); as determined by XPS, the resulting charge states of Ni and Ti in the 2LTO/2LNO sample are in excellent agreement with those obtained by XAS at Ti L_2,3_- and Ni L_2,3_-edges. Moreover, as revealed by the angle-dependent (*ex situ*) XPS (see Supplementary Fig. 4), the pronounced Ni^2+^ peak near the interface of metallic 2LTO/8LNO (see Supplementary Fig. 5) further confirmed the interfacial charge transfer from Ti to Ni sites.

### Electronic reconstruction

**Figure 3:**
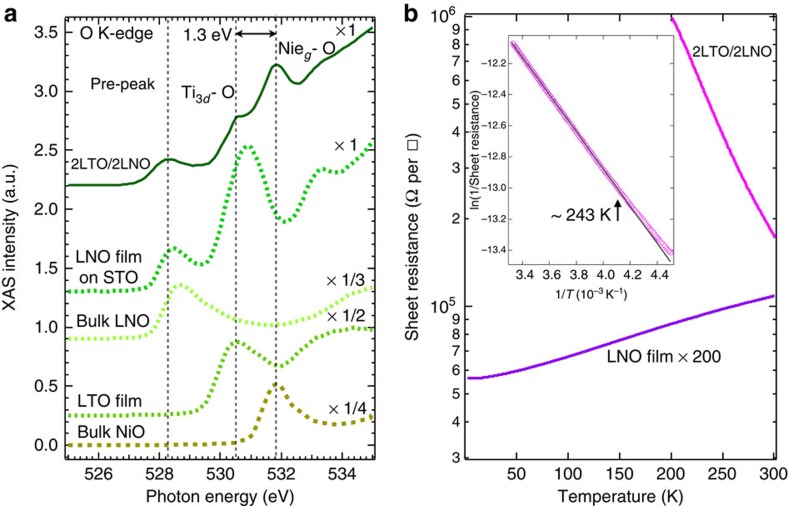
Oxygen K-edge spectra and electrical transport of 2LTO/2LNO. (**a**) Normalized XAS spectra at O K-edge. The black dashed lines indicate the assignments of three key features: the pre-peak (∼528.5 eV) and hybridized Ti 3*d*-O (∼530.5 eV) and Ni *e*_*g*_−O (∼531.8 eV) states by comparing with the spectra of four reference samples LaNi^3+^O_3_ film (10 u.c. on SrTiO_3_ substrate), bulk LaNi^3+^O_3_, LaTi^3+^O_3_ film (20 u.c. on TbScO_3_ substrate^59^) and bulk Ni^2+^O (the data of NiO was adapted from ref. 60). (**b**) Temperature-dependent sheet resistances of the SL 2LTO/2LNO and the reference LaNiO_3_ film (20 u.c.). It is noteworthy that the sheet resistance of LNO film is × 200. Inset: resulting fit of the conductance of 2LTO/2LNO (black solid line) yielding an activation gap *E*_*g*1_∼0.2±0.01 eV (ref. 61).

With the confirmed large interfacial charge transfer from Ti to Ni sites, an important question arises: how does the interfacial charge transfer alter the fundamental physical properties (that is, electronic configuration and band structure) of the 2LTO/2LNO SL? First, we discussed the emergent electronic configuration. As mentioned above, the channels of interfacial charge-transfer Ti *d*^1^+Ni *d*^7^→Ti *d*^0^+Ni *d*^8^ and Ti *d*^1^+Ni *d*^8^L→Ti *d*^0^+Ni *d*^8^ are both open at the interface. Experimentally, owing to the strong hybridization between Ni 3*d* states and oxygen 2*p* states at the Fermi level, XAS at O *K*-edge becomes another important way to probe the charge states of Ni and Ti mixed with ligand holes. As seen in Fig. 3a and Supplementary Fig. 6, in 2LTO/2LNO the oxygen K-edge spectra clearly show a characteristic low-energy pre-peak at ∼528.5 eV, which arises from the ligand holes^18^. In sharp contrast, a direct comparison with the LTO and LNO reference samples immediately shows that the pre-peak at the 2LTO/2LNO interface is strongly suppressed due to the filling oxygen ligand holes with the transferred electrons from Ti sites (also see Supplementary Fig. 6). Based on the absence of the pre-peak feature in the LaTi^3+^O_3_ and Ni^2+^O reference samples, these data imply that the strong suppression of the pre-peak intensity results from the filling of holes on oxygen by the interfacial charge transfer into the Ni *d*-band. As a result, this process induces the formation of the *d*^8^ state and the strong suppression of the *d*^8^L configuration. In addition, we point out at the expected difference between the theory (full charge transfer of one electron)^20^ and the experimental observation of less than one electron transfer, that is, (1−2*δ*) electron; the observed deviation from the full charge transfer in theory is due to the reduced electron filling from unity, 2*δ* of the Ti *d*-band. Combined with the mixed *d*^7^ and *d*^8^L ground state of bulk LNO, this factor results in the observed peculiar electronic configuration of *d*^8^, *d*^7^ and *d*^8^L states, which appears at the interfacial NiO_2_ layer in 2LTO/2LNO.

Next, we discuss the reconstructed band structure at the interface. Because of the observed strong reconstruction of electronic configuration, it is natural to anticipate a similarly strong modification of the band structure near the interface. As predicted by the theory^20^ and illustrated in Fig. 1c, two opening gaps *E*_*g*1_ and Δ_Ti−Ni_ are expected to appear at the interface. First, we estimated a magnitude of the charge gap *E*_*g*1_ by measuring the temperature-dependent electrical transport properties of the 2LTO/2LNO and LNO reference films. As immediately seen in Fig. 3b, the LNO thin film grown at the same conditions as the SL shows a metallic bulk-like behaviour (∼280 Ω per □ at 2 K) from room temperature down to 2 K. In sharp contrast, the SL 2LTO/2LNO displays a highly insulating behaviour with a very large sheet resistance increasing from ∼175 kΩ per □ at 300 K to ∼1 MΩ per □ at 200 K, exceeding the measurement range of the transport setup. This insulating behaviour of the 2LTO/2LNO implies the charge excitations gap opening in 2LTO/2LNO. The resulting fit to the transport data shown in inset of Fig. 3b yields a value of *E*_*g*1_∼0.20±0.01 eV; this is in a accord with the theoretical prediction of the ∼0.4 eV charge-transfer gap^20^.

Next, we estimate the value of the gap Δ_Ti−Ni_ between empty Ti *t*_2*g*_ and Ni *e*_*g*_ bands by measuring XAS of the 2LTO/2LNO film at O K-edge. In a simple ionic model, the configuration of oxygen is O 1*s*^2^2*s*^2^2*p*^6^ and thus the transition 1*s*→2*p* is blocked in the absorption process, because it is a fully occupied 2*p* shell for the O ion. In real materials, however, owing to the strong hybridization the covalent bonding between the transition metal ion and oxygen can introduce a sizable spectral weight of oxygen 2*p* character in the total unoccupied density of states^50^,^51^,^52^,^53^. As a result, O K-edge XAS provides a complimentary way to probe the relative energy position of the TM ion. We also point out that in comparison with the L_2,3_-edge XAS reflecting the absorption process for the specific TM ion, O K-edge provides a convenient way to measure the relative energy position of the unoccupied bands of both TM ions (Ti and Ni) present in the 2LTO/2LNO heterostructure^52^. A direct comparison with the reference samples allows to assign the two peaks at ∼530.5 and ∼531.8 eV shown in Fig. 3a to the hybridized oxygen 2*p* with Ti 3*d* and Ni *e*_*g*_ bands, respectively, and then to extract the value of the Mott gap Δ_Ti−Ni_∼1.3 eV. Assuming that the bandwidth of Ti 3*d*-O and Ni *e*_*g*_-O bands is roughly the same (see Fig. 3a) and with the known value of *E*_*g*1_∼0.2 eV the estimated value of the correlated gap *E*_*g*2_=(*E*_*g*1_+Δ_Ti−Ni_) is ∼1.5 eV, this value is in a remarkable agreement with the theoretically predicted value of ∼1.5 eV (ref. 20). The above observation of the two gaps opening in the excitation spectrum lends strong support to the notion of a strong modification of the band structure at the interface triggered by the redistribution of correlated charges. It is also noted that some additional contributions (for example, disorder effect, electron–electron interactions and charge/spin order)^44^,^54^,^55^ may be involved in the enhanced carrier localization of 2LTO/2LNO. However, their contributions are not dominant in comparison with the large ∼1.5 eV correlated gap of 2LTO/2LNO. As demonstrated by the angle-dependent XPS on 2LTO/8LNO (see Supplementary Fig. 4), the formation of Ni^2+^ accompanied with the strongly suppressed density of states near the Fermi energy level is primarily driven by the interfacial charge transfer from Ti to Ni sites.

### Orbital reconstruction

**Figure 4:**
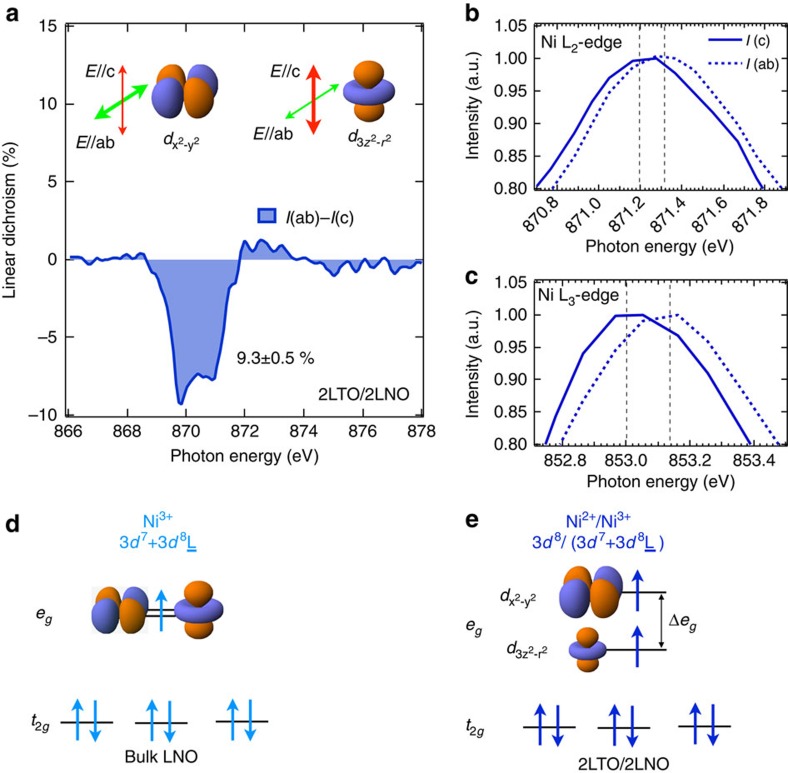
Linear dichroism and reconstructed states. (**a**) XLD (*I*(ab)−*I*(c)) of 2LTO/2LNO interface (extracted from Fig. 2b). Green (in-plane) and red (out-of-plane) arrows label the direction of linear polarization vector (*E*) of the photon. (**b**,**c**) XAS of 2LTO/2LNO at Ni L_2,3_-edge showing the Ni *e*_*g*_ band splitting (∼0.12 for L_2_ and 0.15 eV for L_3_, respectively) of 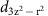 (lower) and 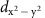 (higher) orbitals. Black dashed lines are guidelines for peak centre positions. (**d**,**e**) Sketch of the engineered electronic, orbital and spin states via interfacial charge transfer for bulk LNO^18^ and SL 2LTO/2LNO, respectively. In SL 2LTO/2LNO, it forms peculiar electronic configuration (Ni 3*d*^7^, 3*d*^8^ and 3*d*^8^L), orbital polarization 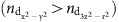 and *e*_*g*_ band splitting (Δ *e*_*g*_∼0.15 eV). Blue arrows indicate the spin configurations of Ni sites.

With the established strongly altered *d*-band filling on Ni and Ti, we investigated the orbital properties of these engineered states on Ni sites. To this end, the orbital polarization has been measured by XLD^16^,^18^,^19^,^56^ on several LNO-based hetrostructures and the result for the LTO/LNO SL is shown in Fig. 4a. Based on the measured electronic state of Ni, one can anticipate that contribution to the XLD signal on Ni L_2_-edge arises largely from the unoccupied Ni 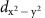 (*I*(ab)) and 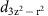 (*I*(c)) states. As illustrated in Fig. 4a, those orbital configurations can be probed with in-plane (*E* || ab, *E* is the polarization vector of the photon) and out-of-plane (*E* || c) linearly polarized photons, respectively. In a good agreement with this expectation, the XLD spectra shown in Fig. 4a show an ample degree of orbital polarization of ∼9.3% at L_2_-edge with the *d*-electron occupancy 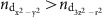 consistent with the first principle calculation prediction of ∼9% (see Calculation details in Methods).

The surprising feature of the XLD data for 2LTO/2LNO is the presence of *e*_*g*_ band splitting (Fig. 4b,c) that was not observed in tensile-strained ultra-thin LNO films or SL (1 u.c.)LNO/(1 u.c.)LaAlO_3_ (refs 18, 19). It is noteworthy that the band splitting is generally estimated by the peak energy shift of XAS (between *I*(ab) and *I*(c)) with linearly polarized photons and the lineshape of XLD (*I*(ab)-*I*(c)) with multiple peak features does not infer the size of the band splitting directly. In the case of 2LTO/2LNO, however, small tensile strain of +1.04% causes the sizable *e*_*g*_ band splitting. Specifically, as seen in inset of Fig. 4b,c, a direct inspection of the energy position for in-plane (∼853.15 eV) and out-of-plane (∼853.0 eV) absorption curves reveal that the out-of-plane absorption is ∼0.15 eV (0.12 eV) lower in energy than the in-plane absorption at Ni L_3_(L_2_)-edge. The difference implies the *e*_*g*_ band splitting Δ*e*_*g*_∼0.15 eV between the states with Ni 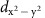 and 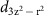 orbital character, as schematically illustrated in Fig. 4d,e. This observation lends a strong support to that recently predicted by theory (see Calculation details in Methods), that both band splitting and orbital polarization arise from the structural distortions at the interface. The apical oxygen atom of out-of-plane Ni–O–Ti bond approaches Ti and leaves away from Ni atoms, whereas the in-plane Ni–O bond length is changed a little and smaller than the out-of-plane Ni–O bond length. This extended out-of-plane Ni–O bond leads to the lowering of Ni 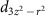 central band energy; on the other hand, it also obviously weakens the hybridization between O 2*p* and Ni 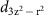 bands with suppressed virtual electron hopping. Therefore, the larger hybridization between O 2*p* and Ni 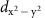 bands results in a higher electron occupancy at higher Ni 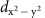
(minority spin) orbital band 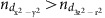, which is very unusual.

In conclusion, by synthesizing the 2LTO/2LNO interface as a prototypical system, we investigated the reconstruction of the local electronic structure on Ni and Ti at the interface between a doped MHI and a charge-transfer metal. Our findings reveal the occurrence of large charge transfer from the Ti to Ni sites across the interface that results in the unusual electronic configurations of Ni 3*d* electrons and lead to the strong modification of the band structure in the vicinity of the interface. In addition, the XLD data show the presence of the large orbital polarization and energy splitting of the Ni *e*_*g*_ band at the vicinity of the interface characteristic of the Jahn–Teller distortion absent in either bulk rare-earth nickelates or other ultra-thin LNO-based heterojunctions. We anticipate that these results will pave the way for follow-up theoretical and experimental work with other important classes of charge-transfer interfaces, to establish a discovery platform for exotic many-body quantum phenomena.

## Methods

### Experiment details

High-quality SLs (2LTO/*n*u.c. LNO) × 10 (*n*=2 and 8, 2LTO/2LNO and 2LTO/8LNO) and reference samples were epitaxially grown by pulsed laser deposition on 5 × 5 × 0.5 mm^3^ (001)-oriented single crystal substrates (LaAlO_3_)_0.3_−(Sr_2_AlTaO_6_)_0.7_ (cubic, *a*=3.87 Å), using a KrF excimer laser operating at *λ*=248 nm and 2 Hz pulse rate with 2 J cm^−2^ fluence. The layer-by-layer growth was monitored by *in-situ* high-pressure reflection high-energy electron diffraction (see Supplementary Fig. 1). To match the growth conditions for both LaTiO_3+*δ*_ and LNO, the SLs 2LTO/2LNO and 2LTO/8LNO were grown under oxygen pressure ∼50 mTorr and the temperature of the substrates was held at 580 °C during the growth. After growth, all samples were cooled at ∼15 °C min^−1^ rate to room temperature keeping oxygen pressure constant. A Mg anode was used for *in-situ* XPS measurements with double-pass cylindrical mirror analysers (STAIB Instruments) at room temperature, whereas for *ex-situ* angle-dependent XPS measurements with a hemispherical electron analyser an Al anode with monochromator (PHI VersaProbe II) was applied. The sheet resistances of the films were measured in van-der-Pauw geometry by Physical Properties Measurement System (Quantum Design) from 300 to 2 K. XAS/XLD with total fluorescence yield mode and X-ray diffraction measurements (room temperature) were carried out at the 4-ID-C and 6-ID-B beamlines, respectively, of the Advanced Photon Source (Argonne National Laboratory).

### Calculation details

Calculated XAS at Ni L_2,3_- and O K-edges of rhombohedral (R-3cH space group) LNO^57^ were carried out with the finite difference method near-edge structure code^58^. In finite difference method near-edge structure calculations we used the full-multiplet scattering (Green function) mode with a large cluster radius of 6 Å around the absorbing Ni atom. The XAS calculations were performed for various Ni 3*d*^*x*^L(7<*x*
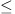
8) configurations of LNO (see Supplementary Fig. 2). To confirm the consistency of the calculated XAS spectra, we also performed XAS calculations using the multi-electron time-dependent DFT (+*U*) with an on-site Coulomb energy on Ni of 6 eV. On the other hand, in the calculation of SL 2LaTiO_3_/2LNO by the DFT+*U* method (*U*_Ni_=6 eV and *U*_Ti_=4 eV), the orbital polarization of unoccupied states is 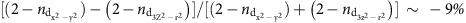, where 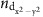 and 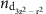 are total electron occupancy (spin up plus spin down) with the 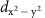 and 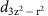 orbital characters of the *e*_*g*_ band, respectively; it is noteworthy that the central energy position of 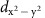 band is higher than that of the 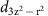 band (personal communication with A.J. Millis and H. Chen).

## Additional information

**How to cite this article:** Cao, Y. *et al.* Engineered Mott ground state in LaTiO_3+*δ*_/LaNiO_3_ heterostructure. *Nat. Commun.* 7:10418 doi: 10.1038/ncomms10418 (2016).

## Supplementary Material

Supplementary Information Supplementary Figures 1-6 and Supplementary References
