# Supplementary material for: Engineered Mott ground state in a LaTiO3+δ/LaNiO3 heterostructure
Source: Nat Commun. 2016 Jan 21;7:10418. doi: 10.1038/ncomms10418 (PMC4735946; doi:10.1038/ncomms10418)
Supplement: Supplementary Information — Supplementary Figures 1-6 and Supplementary References [file ncomms10418-s1.pdf]

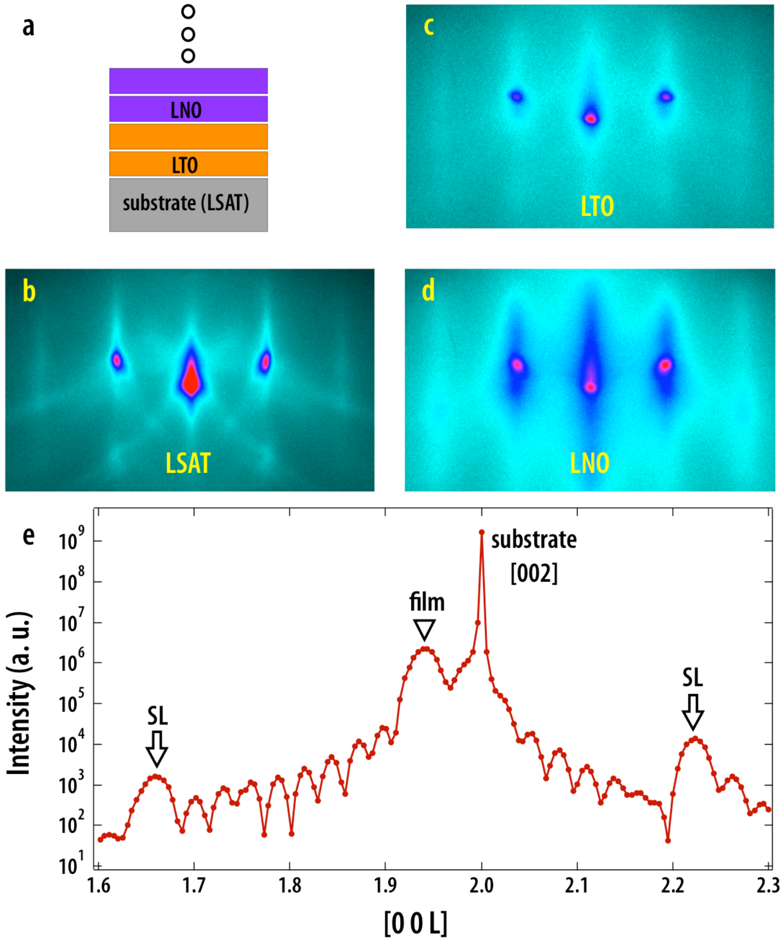

Supplementary Figure 1 I Epitaxy and characterization of 2LTO/2LNO superlattice. (**a**) Schematic view. (**b**)-(**d**) RHEED patterns before [(b)] and during growth [(c) and (d) for LTO and LNO layers, respectively]. (**e**) XRD data (room temperature) of 2LTO/2LNO around [002] peak (pseudocubic notation) with distinct thickness fringes. The black triangle suggests the broad peak of film whereas the arrows mark the superlattice peaks.

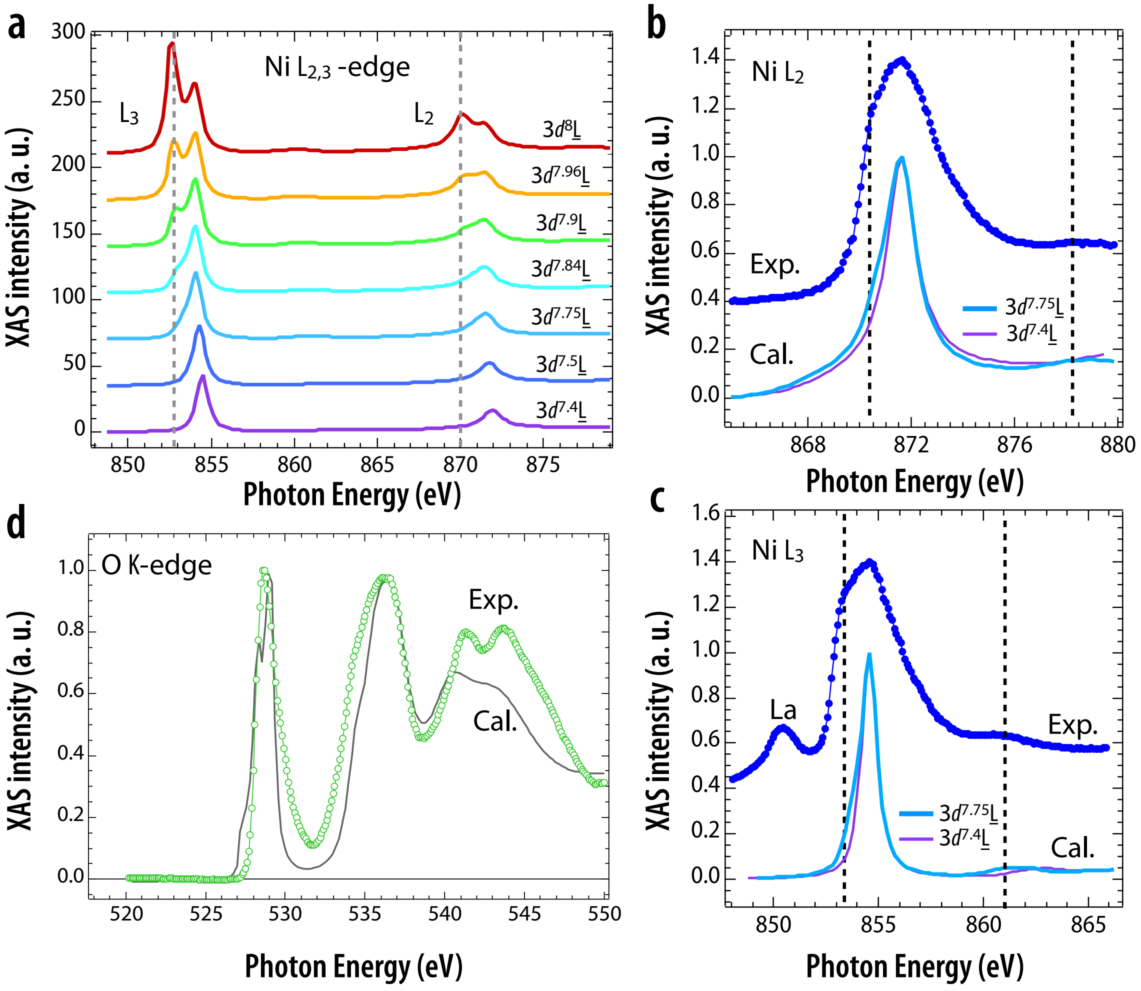

Supplementary Figure 2 | Calculated XAS of bulk LNO with dependence of $3d^{x}\underline{L}$. Here $3d^{x}\underline{L}$ represents an electronic configuration composed of $[(8\text{-}x)\times100]$% $3d^{7}$ and $[(x\text{-}7)\times100]$% $3d^{8}\underline{L}$ for bulk LNO, i.e., Ni $3d^{7.75}\underline{L}$ labels a mixture of 25% $3d^{7}$ and 75% $3d^{8}\underline{L}$ states. (**a**) Ni $L_{2,3}$-edge spectra with $3d^{x}\underline{L}$ from $3d^{7.4}\underline{L}$ to $3d^{8}\underline{L}$. The dashed lines indicate the energy positions of low energy shoulders (or peaks) characterizing the Ni $3d^{8}\underline{L}$ state. (**b**) and (**c**) Comparison of the experimental XAS at Ni $L_{2,3}$-edge with the calculations for x = 7.75 (blue solid lines) and 7.4 (purple solid lines), respectively. As indicated by the dashed lines, calculated XAS with Ni $3d^{7.75}\underline{L}$ shows good agreement with the experimental data for both shoulders (~ 870.5 eV for L_2 and ~ 853.5 eV for L_3, respectively) and bumps (~ 878.25 eV for L_2 and ~ 861 eV for L_3, respectively). (**d**) O K-edge spectra for Ni $3d^{7.75}\underline{L}$. Both Ni $L_{2,3}$- and O K-edges spectra suggest the electronic configurations of bulk LNO is a mixture of ~ 25% d^{7} and ~ 75% $d^{8}\underline{L}$ states.

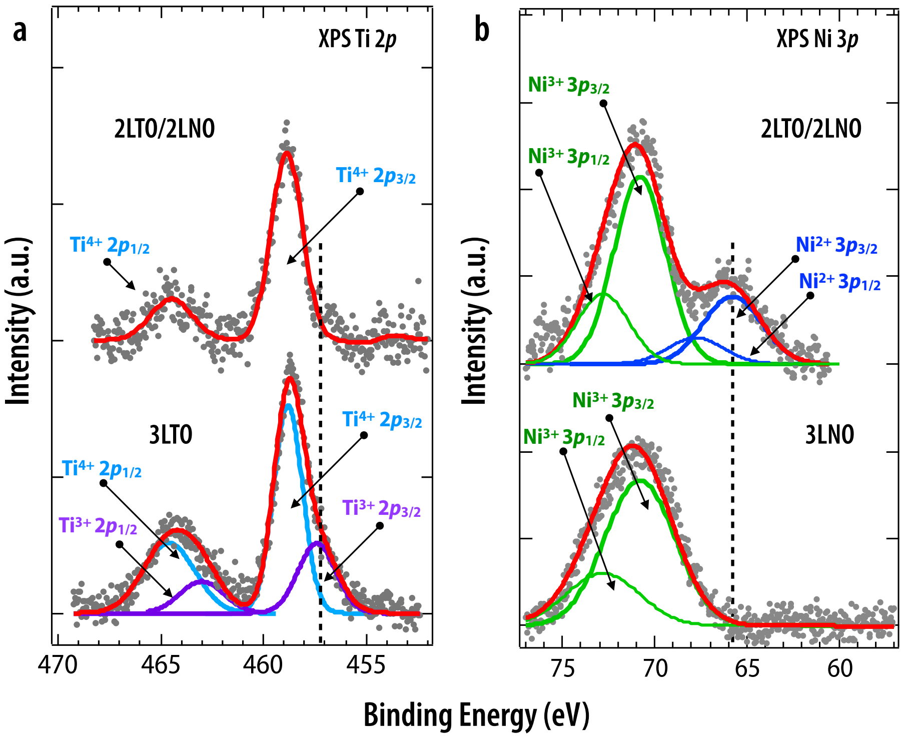

Supplementary Figure 3 | In-situ XPS spectra for Ti 2*p* and Ni 3*p* states. (**a**) Ti 2*p* spectra. In LTO/LNO the valence of Ti is an almost pure Ti^{4+} while it is a mixed Ti^{3+}/Ti^{4+} state in 3 u.c. LTO film synthesized under the same growth conditions. To match the growth condition of LNO, LTO film in our work is oxygen doped, however, which does not affect the charge-transfer picture at the LTO/LNO interface. The determination of the peak area ratio $Ti^{4+}/[Ti^{4+}+Ti^{3+}]$ ~ 0.65 yields the estimation of δ value ~ 0.34 in 3 u.c. $LaTiO_{3+\delta}$ film. (**b**) Ni 3*p* spectra. In LTO/LNO the valence of Ni is a Ni^{2+}/Ni^{3+} state while it is a pure Ni^{3+} state in 3 u.c. film All the data were collected at room temperature in the high vacuum (~ 2×10^{-9} Torr). Grey dots are the experimental data while the solid lines are the fittings of experimental spectra (based on Gaussian functions), which agree well with the recent reports [1, 2].

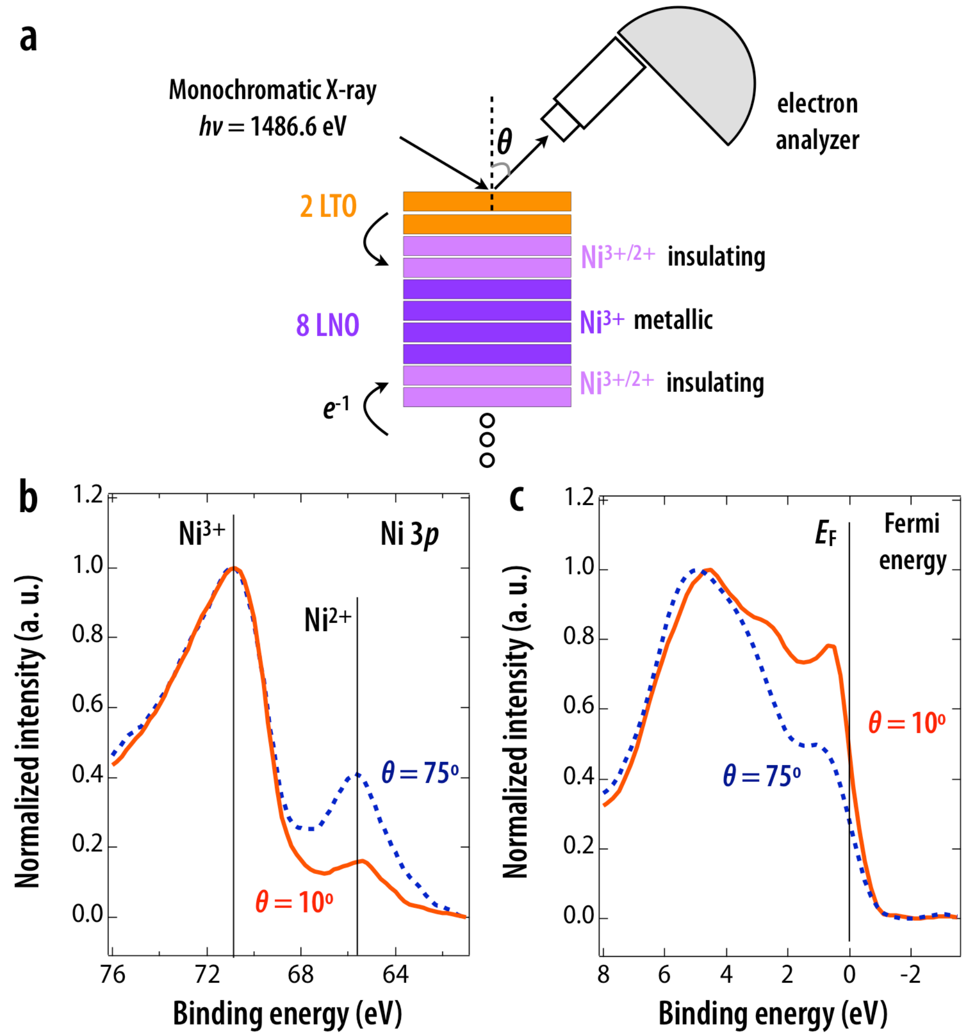

Supplementary Figure 4 I Normalized angle-dependent XPS on metallic 2LTO/8LNO. (**a**) Schematic view of experimental setup. (**b**) Ni 3*p* spectra. The Ni^{2+} peak is pronounced when probing region is near the interface (θ = 75°, blue dashed line). (**c**) Electronic structure near Fermi energy level. The density of states near the Fermi energy level in the interface area (θ = 75°, blue dashed line) is strongly suppressed.

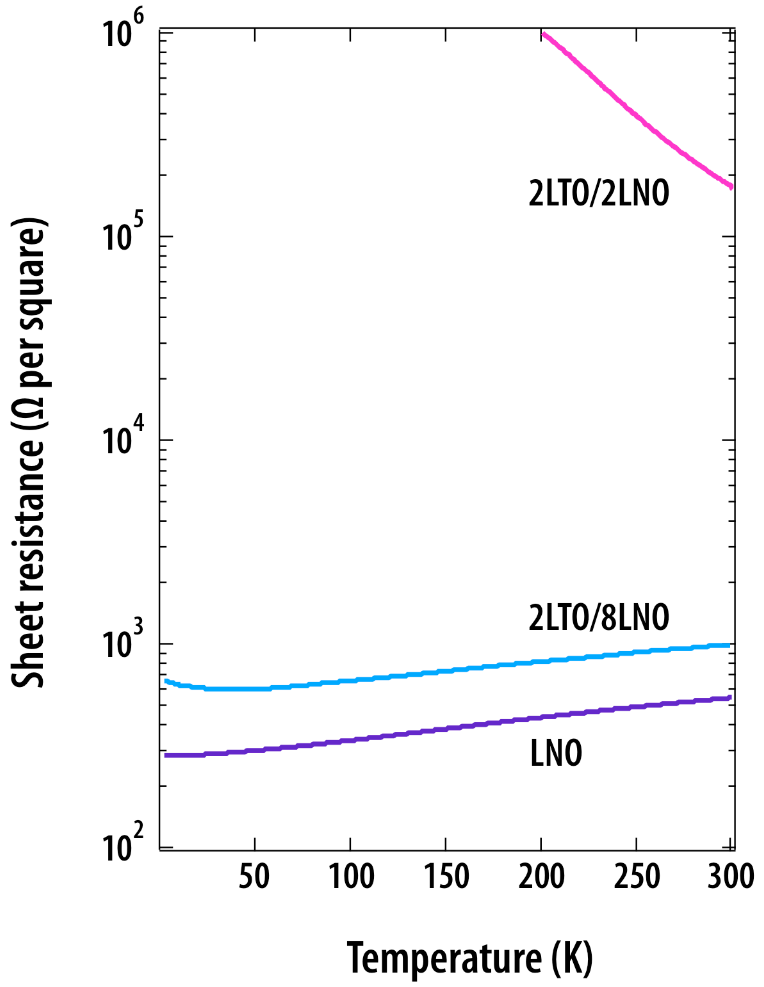

Supplementary Figure 5 | Electrical transport. Sheet resistances of 2LTO/2LNO, 2LTO/8LNO, and 20 u.c. LNO films. Please note the sheet resistance of 2LTO/8LNO is normalized (the contribution of per 20 u.c. LNO layers) to compare with the values of 2LTO/2LNO and 20 u.c. LNO films.

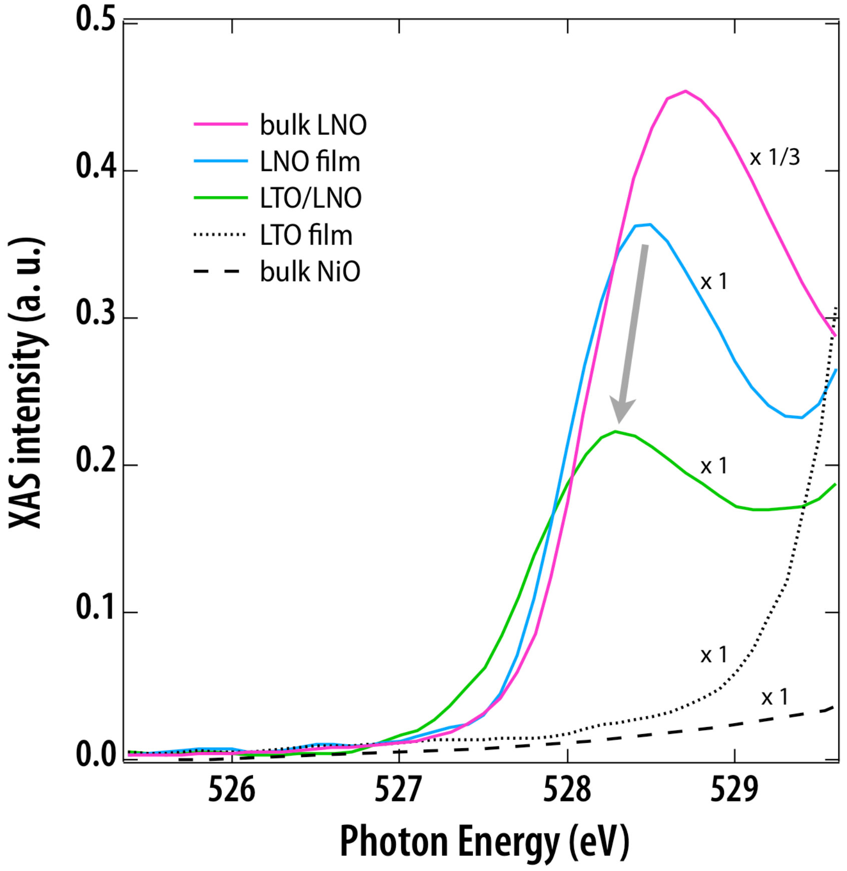

Supplementary Figure 6 l Pre-peak of the normalized XAS at O K-edge. Comparing with the LNO film (10 u.c.) on $SrTiO_3$ substrate (tensile strain, blue solid line), it is seen that the pre-peak intensity of 2LTO/2LNO (green solid line) was strongly suppressed, as shown by the grey arrow, which arises from the filling of ligand holes at oxygen sites by the transferred electrons from Ti sites.

Supplementary Reference:

1. Kareev, M., Cao, Y., Liu, X., Middey, S., Meyers, D. & Chakhalian, J. Metallic conductance at the interface of tri-color titanate superlattices. *Appl. Phys. Lett.* **103**, 231605 (2013).

2. Qiao, L. & Bi, X. Direct observation of Ni^{3+} and Ni^{2+} in correlated $LaNiO_{3-\delta}$ films. *Europhys. Lett.* **93**, 57002 (2011).
